# Supplementary material for: Protocol for the Mindful Student Study: a randomised controlled trial of the provision of a mindfulness intervention to support university students' well-being and resilience to stress
Source: BMJ Open. 2016 Nov 9;6(11):e012300. doi: 10.1136/bmjopen-2016-012300 (PMC5129000; doi:10.1136/bmjopen-2016-012300)
Supplement: supplementary file [file bmjopen-2016-012300supp.pdf]

The Mindful Student Study

Consent form

By agreeing to take part you **declare** that:

Tick box to confirm you have read each statement:

• You have read and understand the **Participant Information Sheet**	
• You have had the opportunity to **ask questions** and had them answered	
• You understand that personal details will remain strictly **confidential** and will be separated from all other information so that researchers work with anonymous data.	
• You understand that your participation is voluntary and that you are **free to withdraw** at any time without giving a reason.	
• You are a **student** at the University of Cambridge	
• You are able to attend at least **seven sessions** of the 8-session mindfulness course	
• **You are NOT**: o Currently suffering from severe periods of anxiety or depression; or o Experiencing severe mental illness such as hypomania or psychotic episodes; or o Recently bereaved or have suffered a major loss (of about six months to a year); or o Experiencing any other serious mental or physical health issue that would impact on your ability to engage with the course.	

By giving the details below you agree to take part in this study:

Name…….

CRSID…….

Signature……..

Date………
